# Efficacy and onset of action of mometasone furoate/formoterol and fluticasone propionate/salmeterol combination treatment in subjects with persistent asthma

**DOI:** 10.1186/1710-1492-7-21

**Published:** 2011-12-07

**Authors:** David I Bernstein, Jacques Hébert, Amarjit Cheema, Kevin R Murphy, Ivan Chérrez-Ojeda, Carlos Eduardo Matiz-Bueno, Wen-Ling Kuo, Hendrik Nolte

**Affiliations:** 1 Division of Immunology, Allergy and Rheumatology, University of Cincinnati College of Medicine, Cincinnati, Ohio, USA; 2 Centre de Recherche Appliquée en Allergie de Québec, Québec, Canada; 3 Alpha Medical Research, Mississauga, Ontario, Canada; 4 Boys Town National Research Hospital, Boys Town, Nebraska, USA; 5 RESPIRALAB Allergy, Respiratory & Sleep Center, Guayaquil, Ecuador; 6 Fundación Salud Bosque, Bogota, Colombia; 7 Merck Sharp & Dohme Corp., a subsidiary of Merck & Co., Inc., Whitehouse Station, NJ USA

**Keywords:** asthma, mometasone furoate/formoterol, fluticasone propionate/salmeterol, noninferiority, onset of action

## Abstract

**Background:**

Mometasone furoate/formoterol (MF/F) is a novel combination therapy for treatment of persistent asthma. This noninferiority trial compared the effects of MF/F and fluticasone propionate/salmeterol (FP/S) combination therapies on pulmonary function and onset of action in subjects with persistent asthma.

**Methods:**

Following a 2- to 4-week run-in period with MF administered via a metered-dose inhaler (MDI) 200 μg (delivered as 2 inhalations of MF-MDI 100 μg) twice daily (BID), subjects (aged ≥12 y) were randomized to MF/F-MDI 200/10 μg BID (delivered as 2 inhalations of MF/F-MDI 100/5 μg) or FP/S administered via a dry powder inhaler (DPI) 250/50 μg (delivered as 1 inhalation) BID for 12 weeks. The primary assessment was change from baseline to week 12 in area under the curve for forced expiratory volume in 1 second measured serially for 0-12 hours postdose (FEV_1 _AUC_0-12 h_). Secondary assessments included onset of action (change from baseline in FEV_1 _at 5 minutes postdose on day 1) and patient-reported outcomes.

**Results:**

722 subjects were randomized to MF/F-MDI (n = 371) or FP/S-DPI (n = 351). Mean FEV_1 _AUC_0-12 h _change from baseline at week 12 for MF/F-MDI and FP/S-DPI was 3.43 and 3.24 L × h, respectively (95% CI, -0.40 to 0.76). MF/F-MDI was associated with a 200-mL mean increase from baseline in FEV_1 _at 5 minutes postdose on day 1, which was significantly larger than the 90-mL increase for FP/S-DPI (*P *< 0.001). The overall incidence of adverse events during the 12-week treatment period that were considered related to study therapy was similar in both groups (MF/F-MDI, 7.8% [n = 29]; FP/S-DPI, 8.3% [n = 29]).

**Conclusions:**

The results of this 12-week study indicated that MF/F improves pulmonary function and asthma control similar to FP/S with a superior onset of action compared with FP/S. Both drugs were safe, improved asthma control, and demonstrated similar results for other secondary study endpoints.

**Trial registration:**

ClinicalTrials.gov: NCT00424008

## Background

Asthma is a chronic inflammatory disorder of the airways that results in recurrent coughing, chest tightness, wheezing, and breathlessness [1]. The first line of therapy to relieve symptoms of persistent asthma is inhaled corticosteroids (ICSs) [1,2]. However, when an ICS alone is unable to control persistent asthma, the Global Initiative for Asthma (GINA) [1] and the National Asthma Education and Prevention Program (NAEPP) [2] guidelines recommend step-up treatment with an ICS combined with a long-acting β_2_-agonist (LABA).

The most recent ICS/LABA combination therapy indicated for the treatment of persistent asthma is mometasone furoate/formoterol delivered via metered-dose inhaler (MF/F-MDI; Dulera^®^/Zenhale^®^, Schering Corporation, a subsidiary of Merck & Co., Inc., Whitehouse Station, NJ). Previous studies have demonstrated the safety and efficacy of MF/F-MDI 100/10 μg twice daily (BID) [3], MF/F-MDI 200/10 μg BID [4], and MF/F-MDI 400/10 μg BID [5] in subjects previously receiving low-, medium-, or high-dose ICS monotherapy, respectively. A long-term safety study [6] also demonstrated that MF/F-MDI 200/10 and 400/10 μg BID were well tolerated with safety profiles similar to equivalent doses of a commonly prescribed ICS/LABA combination, fluticasone propionate/salmeterol (FP/S; Advair^®^/Seritide^®^, GlaxoSmithKline, Research Triangle Park, NC). However, this 1-year safety study was not powered to compare MF/F and FP/S with regard to efficacy. The objective of the current study was to investigate, using an evaluator-blinded, noninferiority design, whether the effects of MF/F-MDI 200/10 μg BID on lung function are noninferior to those of FP/S 250/50 μg BID (delivered via dry powder inhaler [DPI]). In addition, if noninferiority was demonstrated, the trial was designed to investigate whether the onset of action with MF/F-MDI was faster than that with FP/S-DPI.

## Methods

**Figure 1:**
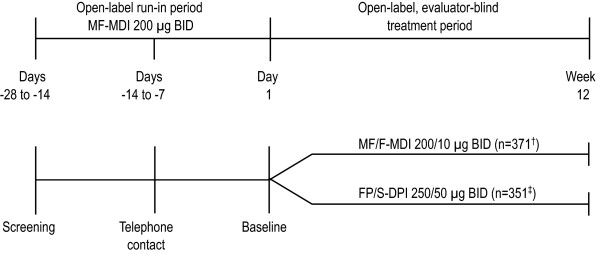
Study Design*. BID = twice daily; DPI = dry powder inhaler; FP/S = fluticasone propionate/salmeterol; MDI = metered-dose inhaler; MF = mometasone furoate; MF/F = mometasone furoate/formoterol. *Doses were delivered via 2 actuations of an MDI (MF 100 μg or MF/F 100/5 μg) or 1 actuation of a DPI (FP/S 250/50 μg) BID. ^†^In the MF/F-MDI 200/10 μg BID treatment group, 42% of subjects discontinued. ^‡^In the FP/S-DPI 250/50 μg BID treatment group, 41% of subjects discontinued.

This was a multicenter, 12-week,^1 ^open-label, evaluator-blinded, active-controlled, noninferiority efficacy and safety trial in subjects (aged ≥12 y) with uncontrolled persistent asthma previously treated with medium-dose ICS with or without a LABA. Following a 2- to 4-week run-in treatment period with MF-MDI 200 μg (delivered as 2 inhalations of MF-MDI 100 μg) BID monotherapy, eligible subjects were randomized in a 1:1 ratio according to a computer-generated randomization schedule to MF/F-MDI 200/10 μg (delivered as 2 inhalations of MF/F-MDI 100/5 μg) BID or FP/S-DPI 250/50 μg (delivered as 1 inhalation) BID for 12 weeks (Figure 1). Study visits were scheduled at screening (days -28 to -14), prebaseline (days -14 to -7), baseline (day 1), and weeks 1, 2, 4, 8, and 12. The first dose of study drug was to be taken in the office under the supervision of a third-party dispenser. Written instructions on the proper use of the MDI or FP/S-DPI were provided to subjects. Subjects assigned to the MDI also used a placebo training inhaler (no DPI placebo training inhaler matching the FP/S-DPI was available for the study). The study protocol and amendments received institutional review board approval and all subjects (or subject's legal representation for those under the age of legal consent) provided written informed consent.

### Patients

Key inclusion criteria were ≥12 years of age; asthma diagnosis for ≥12 months; previous treatment with a medium-dose ICS, either alone or with a LABA, for ≥12 weeks before screening; stable asthma treatment regimen (daily dose unchanged) for ≥2 weeks before screening; history of ≥2 unscheduled asthma-related visits to a physician or emergency department within the past year, or ≥3 unscheduled asthma-related visits within the past 2 years; forced expiratory volume in 1 second (FEV_1_) 60%-90% predicted at screening and baseline; an increase in absolute FEV_1 _of ≥12% and ≥200 mL within 15-20 minutes after administration of short-acting β_2_-agonist (SABA) rescue medication or peak expiratory flow (PEF) variability > 20%; and use of ≥12 inhalations of rescue medication in the final 10 days of the run-in period.

Key exclusion criteria were > 20% change in absolute FEV_1 _between screening and baseline; use of > 8 inhalations per day of a SABA-MDI or ≥2 nebulized treatments per day of 2.5 mg SABA on any 2 consecutive days between screening and baseline; 2 consecutive days before randomization with a decrease in PEF below the run-in stability limit, calculated over the preceding 7 days; clinical deterioration of asthma between screening and baseline that resulted in emergency treatment or hospitalization, or treatment with asthma medications other than a SABA; asthma-related emergency department visit or hospital admission in the past 3 months; current smoker or ex-smoker (ie, smoked in the previous year or had a cumulative smoking history > 10 pack-years).

### Assessments

#### Lung Function

The study was designed to assess the noninferiority of MF/F-MDI 200/10 μg BID compared with FP/S-DPI 250/50 μg BID in their effect on lung function as measured by the change from baseline (mean of 2 predose measurements on day 1) to week 12 (last observation carried forward [LOCF]) in area under the curve (AUC) in FEV_1 _measured serially over 0-12 hours postdose (FEV_1 _AUC_0-12 h_). As a key secondary assessment, the study was also powered to assess whether MF/F-MDI 200/10 μg BID was superior to FP/S-DPI 250/50 μg BID in onset of action (ie, change from baseline in FEV_1 _at 5 minutes postdose on day 1) if lung function noninferiority was demonstrated. Serial spirometry assessments of FEV_1 _were performed after the previous evening's dose at baseline (day 1) and the final visit (week 12) according to the following schedule: 30 minutes and immediately before the subject's morning (AM) dose of study medication; 5, 15, and 30 minutes after the AM dose; and 1, 2, 3, 4, 6, 8, 10, 11, and 12 hours after the AM dose. Additional secondary assessments included changes from baseline in trough FEV_1 _and AM PEF at each visit and week 12 (LOCF).

#### Asthma Control, Quality of Life, and Symptoms

Changes from baseline to week 12 (LOCF) in total Asthma Control Questionnaire (ACQ) [7] score and the proportion of symptom-free (ie, total asthma symptom score = 0 [range, 0 = none to 3 = severe]) days and nights combined were key secondary assessments. Changes from baseline in total Asthma Quality of Life Questionnaire With Standardized Activities (AQLQ[S]) [8] score, 24-hour symptom score, and proportion of nocturnal awakenings due to asthma requiring SABA rescue medication (where baseline was the proportion of nights with nocturnal awakenings [days -7 to 1] before the first treatment dose) were additional secondary assessments.

#### Clinically Judged Asthma Deteriorations

The incidence of clinically judged asthma deteriorations, defined as asthma resulting in emergency treatment, hospitalization, or treatment with additional (excluded) asthma medication (eg, systemic glucocorticoids) were recorded throughout the study.

#### Safety

Safety was assessed by monitoring adverse events (AEs), clinical laboratory tests, physical examinations (including oropharyngeal examination), vital signs, and electrocardiogram (ECG) recordings throughout the study.

### Statistical Analyses

Assuming a dropout rate of 5%, a sample size of 332 subjects for each treatment group was to be enrolled to ensure that 315 subjects would be available for the test of noninferiority based on the change from baseline to week 12 (LOCF) in FEV_1 _AUC_0-12 h _at 80% power, assuming a standard deviation of 6.7 L × h. Noninferiority for the primary variable was achieved when the lower bound of a 2-sided 95% confidence interval of the treatment difference (MF/F-MDI - FP/S-DPI) exceeded -1.5 L × h in the change from baseline to week 12 (LOCF) in FEV_1 _AUC_0-12 h_. The selected lower bound of -1.5 L × hr was one-half of an estimated treatment difference of an active treatment versus placebo (3.1 L × h).

Changes from baseline in all assessments were analyzed by analysis of covariance, using treatment and study site as fixed effects and the corresponding baseline value for that assessment as a continuous covariate.

## Results

### Demographics and Disposition

A total of 722 subjects were randomized to receive MF/F-MDI 200/10 μg BID (n = 371) or FP/S-DPI 250/50 μg BID (n = 351). Demographic and baseline characteristics were balanced across the 2 treatment groups. Most subjects were female (459/722; 64%) and white (624/722; 86%; Table 1); mean age was 44.9 years. Median duration of asthma was 12 years. Before participation in the study, most subjects were receiving 1 of the following: budesonide (monotherapy or in combination), beclomethasone (monotherapy), or fluticasone (monotherapy or in combination). Subjects had moderate persistent asthma that was uncontrolled after the MF 200-μg BID run-in period, based on FEV_1 _(ie, 60%-80% predicted) and ACQ (ie, score ≥1.5) findings as related to definitions from the NAEPP [2].

**Table 1:** Subject Demographics and Baseline Characteristics

Demographic or Characteristic	MF/F-MDI 200/10 μg BID (n = 371)	FP/S-DPI 250/50 μg BID (n = 351)
Sex, n (%)		
Female	239 (64)	220 (63)
Race, n (%)		
White	323 (87)	301 (86)
Mean age, y (range)	44.8 (12-82)	45.1 (12-80)
Duration of asthma, y		
Mean	15.0	15.9
Median	11.0	13.0
Prior ICS use, n (%)*	225 (61)	188 (54)
Prior ICS/LABA use, n (%)*		
Budesonide/formoterol	59 (16)	59 (17)
Fluticasone/salmeterol	104 (28)	115 (33)
Mean FEV_1 _at baseline		
L	2.30	2.37
Percentage predicted	73.8	74.4
Mean total ACQ score^†^	1.80	1.80
Mean total AQLQ(S) score^‡^	5.14	5.14

ACQ = Asthma Control Questionnaire; AQLQ(S) = Asthma Quality of Life Questionnaire With Standardized Activities; BID = twice daily; DPI = dry powder inhaler; FEV_1 _= forced expiratory volume in 1 s; FP/S = fluticasone propionate/salmeterol; ICS = inhaled corticosteroid; LABA = long-acting β_2_-agonist; MDI = metered-dose inhaler; MF/F = mometasone furoate/formoterol.

*Subjects could have received multiple types of ICS monotherapy and combination ICS/LABA therapy in the 3 months before screening.

^†^ACQ score based on a 7-point scale that ranged from 0 (best asthma control) to 6 (worst asthma control).

^‡^AQLQ(S) score based on a 7-point scale that ranged from 1 (worst quality of life) to 7 (best quality of life)

The percentages of subjects who discontinued the trial through week 12 were 42% and 41% in the MF/F-MDI 200/10 μg BID and FP/S-DPI 250/50 μg BID treatment groups, respectively. Administrative reasons^2 ^were the most common cause of subject discontinuation in both treatment groups (25.6% and 24.2%). The mean times to administrative discontinuation were 76.0 days in the MF/F-MDI and 80.2 days in the FP/S-DPI groups, close to the 85-day treatment duration scheduled for a 12-week study. Fully 81.3% of subjects (579/712) remained in the trial long enough to qualify for the Week 12 observed cases evaluation window, compared to the primary endpoint last-observation-carried-forward into Week 12, which includes those who discontinued earlier. Discontinuation as the result of an AE represented only a small proportion of subjects (approximately 2%) in each treatment group, while discontinuation for treatment failure represented 5% of subjects in each treatment group.

### Lung Function

**Figure 2:**
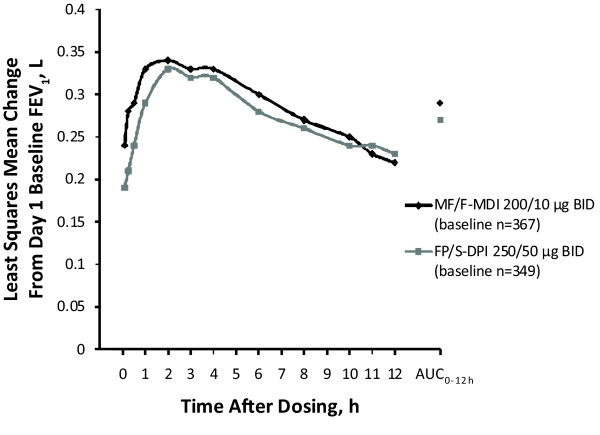
**Serial FEV_1 _(0-12 h) and Standardized FEV_1 _AUC_0-12 h _at Week 12 (LOCF)**. AUC = area under the curve; BID = twice daily; DPI = dry powder inhaler; FEV_1 _= forced expiratory volume in 1 second; FP/S = fluticasone propionate/salmeterol; LOCF = last observation carried forward; MDI = metered-dose inhaler; MF/F = mometasone furoate/formoterol.

At week 12 (LOCF), FEV_1 _AUC_0-12 h _for MF/F-MDI and FP/S-DPI was 3.43 vs 3.24 L × h, respectively (95% CI, -0.40 to 0.76), indicating that MF/F-MDI was not inferior to FP/S-DPI; noninferiority was also demonstrated on day 1 (3.66 vs 3.29 L × h [95% CI, -0.11 to 0.84]) and week 12 (3.45 vs 3.33 L × h [95% CI, -0.56 to 0.79]). Mean FEV_1 _AUC_0-12 h _values at week 12 (LOCF) corresponded to standardized increases from baseline of 0.29 L (12.7%) for MF/F-MDI and 0.27 L (12.1%) for FP/S-DPI when averaged across the 12-hour serial spirometry evaluation period (Figure 2).

**Figure 3:**
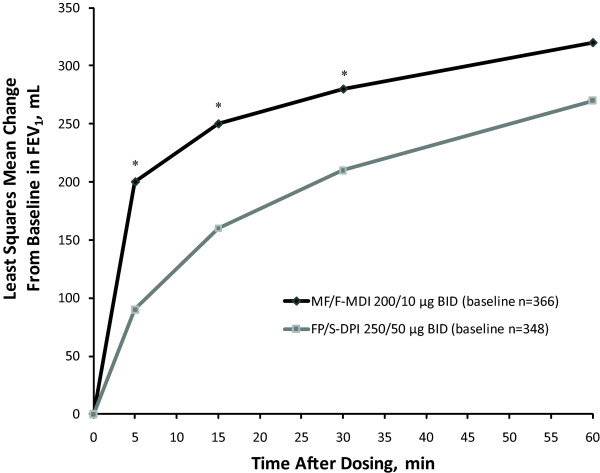
**Onset of Action: Serial FEV_1 _(0-1 h) at Day 1**. BID = twice daily; DPI = dry powder inhaler; FEV_1 _= forced expiratory volume in 1 second; FP/S = fluticasone propionate/salmeterol; MDI = metered-dose inhaler; MF/F = mometasone furoate/formoterol. *P < 0.001.

Analysis of onset-of-action characteristics revealed that the effect of MF/F-MDI occurred significantly faster than the effect of FP/S-DPI (Figure 3). At 5 minutes postdose (the first scheduled measurement) on day 1 of the study, MF/F-MDI was associated with a 200-mL least-squares (LS) mean increase from baseline in FEV_1 _versus a 90-mL increase with FP/S-DPI (*P *< 0.001). When adjusting for multiplicity (using *P *< 0.0125), the rapid, persistent effect of MF/F-MDI on FEV_1 _was significantly greater than the effect of FP/S-DPI for all time points measured up to 30 minutes postdose (*P *< 0.001; Figure 3).

Baseline LS mean trough FEV_1 _values were 2.31 and 2.39 L in the MF/F-MDI and FP/S-DPI groups, respectively. Changes from baseline in trough FEV_1 _were not significantly different between groups at week 12 (LOCF) (0.14 and 0.17 L, respectively) or any time during the 12-week treatment period.

Both groups had similar baseline LS mean AM PEF values (MF/F-MDI, 360.3 L/min; FP/S-DPI, 363.3 L/min), and both treatments resulted in stable and positive AM PEF changes from baseline. There were no significant differences between treatments in change from baseline LS mean AM PEF at any week or week 12 (LOCF) (MF/F-MDI, 21.3 L/min [6.9%]; FP/S-DPI, 23.0 L/min [7.9%]).

### Asthma Control, Quality of Life, and Symptoms

**Figure 4:**
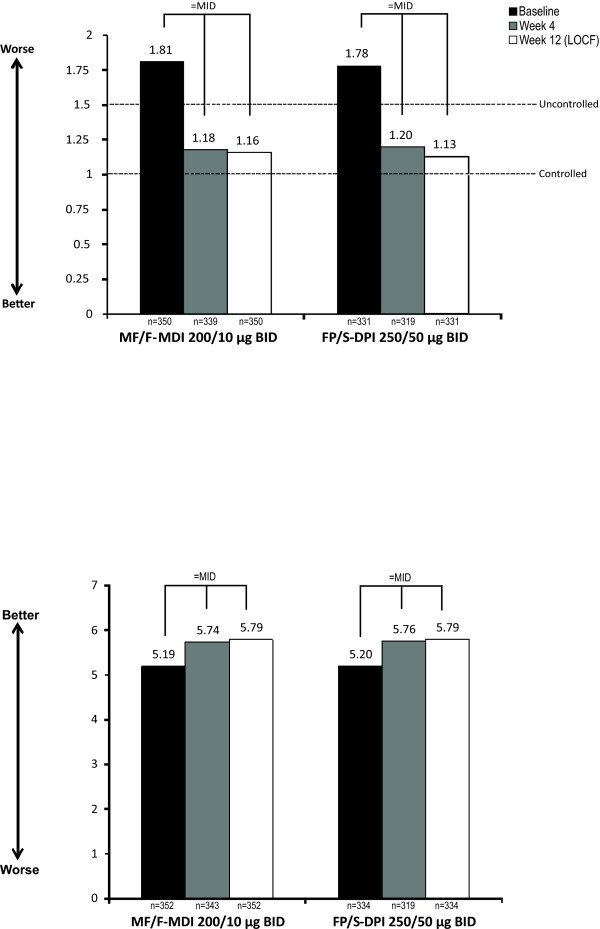
Mean Total ACQ* (top) and AQLQ(S)^† ^(bottom) Scores. ACQ = Asthma Control Questionnaire; AQLQ(S) = Asthma Quality of Life Questionnaire With Standardized. Activities; BID = twice daily; DPI = dry powder inhaler; FP/S = fluticasone propionate/salmeterol; LOCF = last observation carried forward; MDI = metered-dose inhaler; MF/F = mometasone furoate/formoterol; MID = minimally important difference. *ACQ score based on a 7-point scale that ranged from 0 (best asthma control) to 6 (worst asthma control). ^†^AQLQ(S) score based on a 7-point scale that ranged from 1 (worst quality of life) to 7 (best quality of life).

At week 4 and week 12 (LOCF), MF/F-MDI was noninferior to FP/S-DPI in LS mean total ACQ and AQLQ(S) score changes from baseline (Figure 4). In both groups, ACQ scores improved to levels that were below the uncontrolled threshold. In addition, ACQ and AQLQ(S) score changes achieved a minimally important difference (MID; ≥0.5) [9,10] in both groups.

Both groups had the same LS mean baseline proportion of nights with nocturnal awakenings due to asthma that required the use of a SABA (0.23). Both treatments reduced this proportion by > 65% at week 12 (LOCF) (MF/F-MDI, -0.14 [-65.5%]; FP/S-DPI, -0.16 [-69.8%]); there was no significant difference between the groups.

Total LS mean 24-hour asthma symptom scores were similar between the groups (MF/F-MDI, 1.87; FP/S-DPI, 1.89). Both treatments improved (ie, reduced) LS mean symptom scores by ≥40% at week 12 (LOCF) (MF/F-MDI, -0.82 [-40.0%]; FP/S-DPI, -0.91 [-49.9%]); there was no significant difference between the groups.

**Figure 5:**
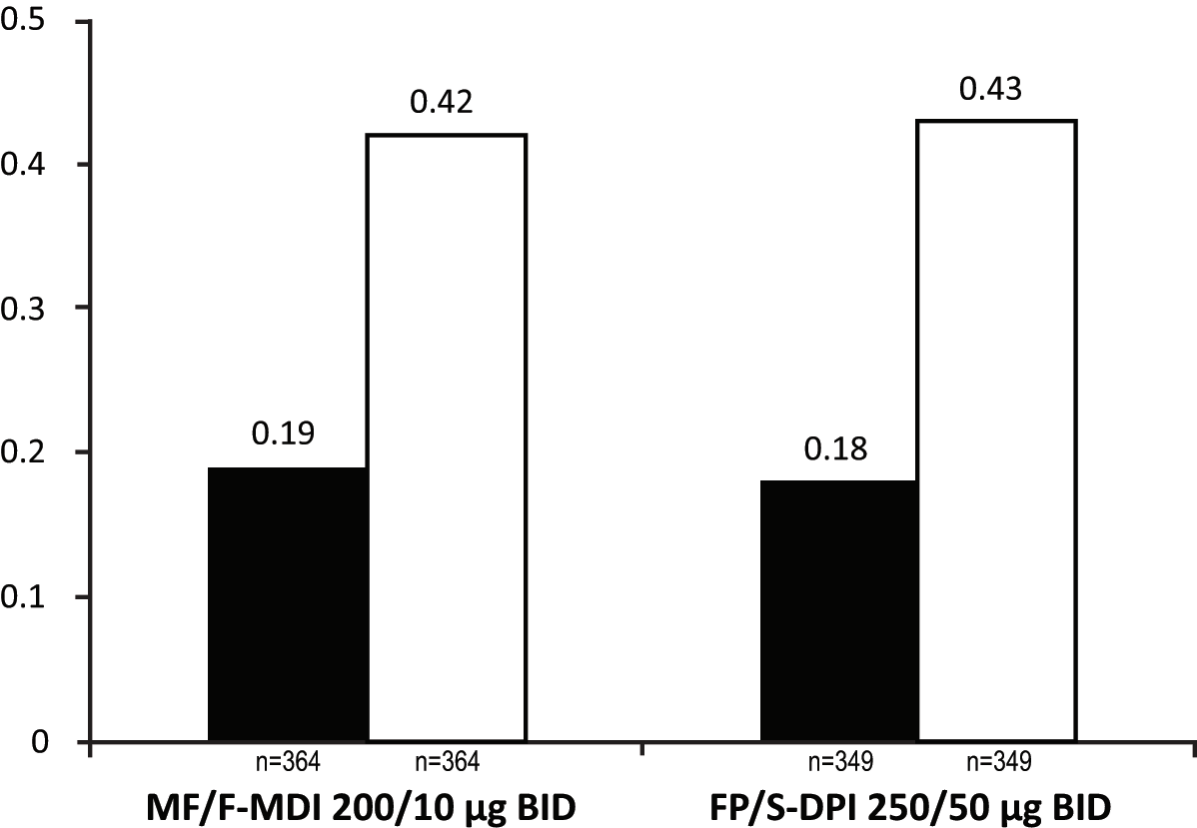
**Proportion of Symptom-Free Days and Nights**. BID = twice daily; DPI = dry powder inhaler; FP/S = fluticasone propionate/salmeterol; LOCF = last observation carried forward; MDI = metered-dose inhaler; MF/F = mometasone furoate/formoterol.

MF/F-MDI was found to be noninferior to FP/S-DPI in the proportion of symptom-free days and nights; both treatment groups demonstrated improvements from baseline (Figure 5).

### Clinically Judged Asthma Deteriorations

The percentage of subjects who experienced a clinically judged asthma deterioration (ie, a deterioration of asthma requiring further treatment; see methods) was the same in both treatment groups (MF/F-MDI, 5.7% [n = 21]; FP/S-DPI, 5.7% [n = 20]).

### Safety

The incidence of AEs during the 12-week treatment period that were considered related to study therapy was similar in both groups (MF/F-MDI, 7.8% [n = 29]; FP/S-DPI, 8.3% [n = 29]). The most common treatment-related AEs were dysphonia (MF/F-MDI, 1.6% [n = 6]; FP/S-DPI, 2.8% [n = 10]), headache (MF/F-MDI, 0.8% [n = 3]; FP/S-DPI, 0.9% [n = 3]), oropharyngeal pain (MF/F-MDI, 1.1% [n = 4]; FP/S-DPI, 0.6% [n = 2]), and oropharyngeal candidiasis (MF/F-MDI, 0.5% [n = 2]; FP/S-DPI, 0.6% [n = 2]). Serious AEs occurred infrequently (MF/F-MDI, 1.3% [n = 5]; FP/S-DPI, 1.4% [n = 5]); only 1 serious AE (ventricular extrasystoles, MF/F-MDI) was considered probably treatment related. No life-threatening AEs were reported, and no subjects died during the study. No clinically relevant changes in laboratory values, mean vital signs, or ECG measurements were observed.

## Discussion

This study in adult and adolescent subjects with persistent asthma uncontrolled on medium-dose ICS monotherapy demonstrated that treatment with MF/F-MDI 200/10 μg BID improved lung function and other patient-reported outcomes similar to FP/S-DPI 250/50 μg BID with an onset of action superior to FP/S-DPI. Although stable lung function was demonstrated during the open-label run-in period, subjects were still uncontrolled at baseline (ie, total ACQ score ≥1.5), indicating that medium-dose MF alone was suboptimal for treating this subject population. Two key observations can be taken from the lung function results regarding the efficacy of MF/F over 12 weeks of treatment. First, treatment with MF/F-MDI 200/10 μg BID was noninferior compared with FP/S-DPI 250/50 μg BID based on FEV_1 _AUC_0-12 h _at week 12 (LOCF). Second, a significant (≥200 mL from baseline) bronchodilator effect of the MF/F combination was observed as early as 5 minutes postdose, which was superior to FP/S through 30 minutes on day 1. This result was expected given the known characteristics of these ICS/LABA combination constituents. Previous studies have demonstrated the efficacy of MF [11-14] and FP [15-17] monotherapy in improving lung function outcomes in patients with asthma. Although LABA monotherapy should not be used to treat asthma [18], formoterol and salmeterol have also been shown to be effective in improving lung function outcomes [19]. However, formoterol is associated with a faster onset of action than salmeterol [20,21]. As such, it does not seem surprising that MF/F was associated with a faster onset of action than FP/S in the current study.

As recently reviewed by Murphy and Bender [22], several surveys have indicated that rapid onset of action is a highly desirable attribute of asthma therapy from a patient perspective. The perception that controller medication is working immediately is a strong predictor of overall treatment satisfaction and may lead to improved medication adherence [22]. However, although the present study demonstrated greater improvements in pulmonary function 5-30 minutes postdose for the MF/F-MDI group compared with the FP/S-DPI group, it did not measure corresponding symptom scores during that time period. Thus, the clinical relevance of the faster onset of action with MF/F-MDI compared with FP/S-DPI has not been determined.

At week 12 (LOCF), a MID in total ACQ score was achieved in both treatment groups and both treatments also shifted patients to the "controlled" [23] ACQ score range. Other secondary efficacy results (eg, improvements in the AQLQ[S] total score) further indicated that MF/F-MDI 200/10 μg BID is noninferior to FP/S-DPI 250/50 μg BID and that both treatments improved asthma outcomes compared with baseline measurements.

A review of the safety data indicated that both treatments were well tolerated. The number and type of AEs reported in this study were not unexpected and did not raise any new safety concerns with regard to MF/F-MDI or FP/S-DPI. The most common treatment-related AE reported was dysphonia, which is a known side effect of ICS treatment [24].

This study was originally scheduled for 1 year and was stopped early due to protracted enrollment period, which resulted in a longer than expected study duration. At the termination of the study, 12-week data were available for 81.3% of patients (579 of the 712 patients originally enrolled in the study), and 40% of noncompletion occurred after the termination of the study. Therefore, most of these discontinuations were a product of early trial termination. Yet because the mean times to administrative discontinuation were approximately 80 days in each treatment group--close to the 85-day treatment duration scheduled for a 12-week study--and because a large proportion of subjects (81.3%) remained in the trial long enough to qualify for the Week 12 observed cases evaluation window, the integrity of the population for noninferiority evaluation was not compromised. Similarly, the proportions of discontinuations owing to reasons other than administrative are similar to those for other MF/F-MDI studies [3-5].

This trial was of 12 weeks' duration and, as such, does not provide insight into the longer-term efficacy and safety of MF/F-MDI. The longer-term safety of MF/F-MDI has been demonstrated by Maspero et al [6]. The trial was of an open-label design and is, therefore, subject to the typical limitations inherent with open-label study. However, an open-label randomized design with frequent, uniform study visits and robust clinical endpoints, such as that used for this study, can be appropriate for comparing similar treatments to determine noninferiority. In this context, it is of interest to note the primary analysis of FEV_1 _AUC at endpoint favored MF/F-MDI by 20 mL over FP/S-DPI in a design setting that usually favors the comparator marketed product. While the study was open-label, subjects were expected to be more familiar with the treatment administration of FP/S-DPI than the use of the MF/F-MDI test product.

An important consideration with this noninferiority design was the demonstration of assay sensitivity. Thus, the subject's own treatment was discontinued at screening, and a run-in period was carried out during which subjects had to demonstrate a degree of asthma deterioration on MF monotherapy. The results show that the study population was appropriately treated with combination therapy and provided assurance that the study population was sensitive enough to detect a potential difference between the MDI and DPI formulations by demonstrating that subjects were responsive to changes in treatment. Therefore, the results indicate that subjects not well controlled on ICS monotherapy will improve lung function and asthma control to the same degree when treated with either combination. It is also important to note that the current study was adequately powered to test for superiority of the onset of action endpoint, and previous trials [3-5] have demonstrated the superiority of MF/F compared with placebo, MF, and/or formoterol for various efficacy assessments.

## Conclusion

In conclusion, the results of this study indicated that MF/F-MDI 200/10 μg BID is noninferior based on FEV_1 _AUC_0-12 h _and superior in onset of action to FP/S-DPI 250/50 μg BID over 12 weeks of treatment. Both combinations were safe, improved asthma control, and demonstrated similar results for other secondary study endpoints.

## Competing interests

David I. Bernstein has received honoraria as a member of Merck & Co., Inc. advisory boards, served as a consultant for GlaxoSmithKline, and has been a clinical investigator for clinical trials sponsored by Merck & Co, Inc. and GlaxoSmithKline. Jacques Hébert has received speaker/consultancy fees and research support from Merck Canada. Amarjit Cheema has received speaker fees and research support from Merck & Co., Inc. Kevin R. Murphy has received speaker/consultancy fees and research support from AstraZeneca, Boehringer Ingelheim, Dey, Merck& Co., Inc, Genentech, GlaxoSmithKline, and Novartis. Ivan Chérrez-Ojeda has received lecture fees from Merck & Co., Inc. Carlos Eduardo Matiz Bueno's spouse is an employee of Merck & Co., Inc. Wen-Ling Kuo and Hendrik Nolte are employees of Merck Sharp & Dohme Corp., a subsidiary of Merck & Co., Inc., Whitehouse Station, NJ.

## Authors' contributions

All authors made substantial contributions to the study conception and design, or acquisition of data, or analysis and interpretation of data; have been involved in drafting the manuscript and/or revising it critically; and have given final approval of the published version.
